# Role for Maternal Asthma in Severe Human Metapneumovirus Lung Disease Susceptibility in Children

**DOI:** 10.1093/infdis/jiaa019

**Published:** 2020-01-22

**Authors:** Romina Libster, Ignacio Esteban, Alejandra Bianchi, Luciano Alva Grimaldi, Karina Dueñas, Andrea Sancillo, Andrea Rodriguez, Fernando Ferrero, Katherine Stein, Patricio L Acosta, Fausto M Ferolla, Eduardo Bergel, Mauricio T Caballero, Fernando P Polack, for the INFANT Respiratory Network, Gustavo Pellegrino, Guadalupe Fernandez Gago, Cecilia Pozzolo, Laura Castro, Rodrigo Egues Almeida, Beatriz Rebec, Mariela Gonzalez, Mariel Calvo, Julieta Henrichsen, Celina Nocito, Guillermo Barbero, Juan Ves Losada, Angel Bonina, Edgardo Flamenco, Alberto Rodriguez Perez, Alicia Kobylarz, Mirta Raggio, Graciela Schavlosky, Adriana Caria, Edgar Barboza, Gustavo Sastre

**Affiliations:** 1 Fundación INFANT, Buenos Aires, Argentina; 2 Consejo Nacional de Investigaciones Científicas y Técnicas, Buenos Aires, Argentina; 3 Hospital de Pediatría Prof. Dr. Juan P. Garrahan, Buenos Aires, Argentina; 4 Hospital Zonal General de Agudos “Lucio Melendez,” Almirante Brown, Argentina; 5 Hospital Interzonal General de Agudos “Evita,” Lanus, Argentina; 6 Hospital Zonal General de Agudos Descentralizado “Evita Pueblo,” Berazategui, Argentina; 7 Hospital General de Niños “Pedro de Elizalde,” Buenos Aires, Argentina

**Keywords:** human metapneumovirus, burden of illness, children, lower respiratory tract infection, risk factors, maternal asthma

## Abstract

**Background:**

Severity of human metapneumovirus (hMPV) lower respiratory illness (LRTI) is considered similar to that observed for respiratory syncytial virus (RSV). However, differences in severity between these pathogens have been noted, suggesting the degree of illness may vary in different populations. Moreover, a potential association between hMPV and asthma also suggests that hMPV may preferentially affect asthmatic subjects.

**Methods:**

In a population-based surveillance study in children aged <2 years admitted for severe LRTI in Argentina, nasopharyngeal aspirates were tested by RT-PCR for hMPV, RSV, influenza A, and human rhinovirus.

**Results:**

Of 3947 children, 383 (10%) were infected with hMPV. The hospitalization rate for hMPV LRTI was 2.26 per 1000 children (95% confidence interval [CI], 2.04–2.49). Thirty-nine (10.2%) patients infected with hMPV experienced life-threatening disease (LTD; 0.23 per 1000 children; 95% CI, .16–.31/1000), and 2 died (mortality rate 0.024 per 1000; 95% CI, .003–.086). In hMPV-infected children birth to an asthmatic mother was an increased risk for LTD (odds ratio, 4.72; 95% CI, 1.39–16.01). We observed a specific interaction between maternal asthma and hMPV infection affecting risk for LTD.

**Conclusions:**

Maternal asthma increases the risk for LTD in children <2 years old hospitalized for severe hMPV LRTI.

## Teaser

In a population-based surveillance study in children aged <2 years admitted for severe lower respiratory tract illness in Argentina including more than 3900 patients, maternal asthma was associated with higher risk for life-threatening disease in human metapneumovirus infections.

Lower respiratory tract illness (LRTI) due to respiratory viruses is the leading cause of hospitalization in infants and young children worldwide [1, 2]. Human metapneumovirus (hMPV) is a frequent pediatric pathogen, associated with 5%–25% of all cases of LRTI [3–6]. As with other pathogens, its burden is higher in developing countries and low-income populations [7–9].

The clinical presentation and virulence of hMPV LRTI in infants and young children resembles that of other viruses, including respiratory syncytial virus (RSV), being also capable of eliciting life-threatening disease (LTD) [10, 11]. That is why, in the context of the changing landscape of new interventions against RSV, it is important to understand how hMPV disease behaves in children and identify novel, preventable risk factors for severe disease.

Previously known risk factors for poor clinical evolution in children hospitalized for hMPV LRTI, such as prematurity, congenital heart disease, immunodeficiency, and neuromuscular disease, are not hMPV specific [3, 12–15]. Considering that the pattern of T-helper 2 (Th2) cytokines observed in respiratory secretions during hMPV LRTI may sometimes resemble that of individuals with asthma (including eosinophilic inflammation, higher interleukin-4 [IL-4] and IL-5 levels in mouse models [16], and induction of thymic stromal lymphopoietin in human airway cells [17, 18]) we speculated that a history of asthmatic predisposition could specifically modify severity of hMPV disease in children.

## METHODS

### Study Design

We conducted a prospective, multicenter active surveillance study to characterize the burden of severe LRTI in children from a low-income region in Buenos Aires, Argentina from 2011 to 2013. Details of the population are provided elsewhere [19]. The study was held in 12 public hospitals, from a geographically defined low-income region in the Buenos Aires suburbs, that provide care to an estimated population of 56 560 children younger than 2 years who lacked medical insurance [20]. Previous studies in this population examined RSV LRTI severity and the role of cytokines in human rhinovirus (hRV) infections [19, 21–25]. The study was approved by institutional review boards at each participating hospital, the state of Buenos Aires, and Vanderbilt University. All participating families signed an informed consent to join the study.

Eligibility criteria included infants and children younger than 2 years admitted for severe LRTI, defined as the sudden onset of cough, wheezing, retractions and/or crackles, with or without fever, and an oxygen saturation <93% when breathing room air or the need for O_2_ support on arrival to emergency rooms. LTD was defined as O_2_ saturation ≤87% on admission, requirement for mechanical ventilation, and/or admission to the intensive care unit.

### Demographic and Clinical Information

Information on demographic, epidemiological, and clinical status was recorded from all participating patients from the time of admission until discharge using specifically designed questionnaires. In the study, we evaluated risk factors for hospitalization including prematurity (<37 weeks’ gestation at birth), age, sex, lack of breastfeeding, malnutrition, house and floor materials, smoking at home, sources of heating, crowding (defined as more than 3 persons/room), parent’s education, and availability of sewage system. Maternal asthma was defined as a physician diagnosis of asthma in the mother before infant enrollment.

### Laboratory Tests

Nasopharyngeal aspirates were collected at the time of admission in patients from consenting families. Samples were tested in duplicate by real-time reverse transcriptase polymerase chain reaction (RT-PCR) for hMPV. Samples were also tested for influenza A viruses, RSV, and hRV, as previously described [19].

### Statistical Analysis

Rates of severe and life-threatening hMPV disease were calculated by dividing the number of patients hospitalized with hMPV severe respiratory infection by the estimated census annual population in the selected area [20]. Student *t* test and *χ*^2^ were used to compare children’s clinical and epidemiological characteristics when appropriate. For the evaluation of risk factors associated with hMPV LTD, we used a multivariable logistic regression model. Covariates selected a priori and with a *P* value < .1 in univariate analysis were included in the model and analyzed in a stepwise forward logistic regression. A *P* value of less than .05 was considered statistically significant. Statistical analyses were performed using the Stata package for IBM-PC (Stata Corp).

## RESULTS

### Burden of Human Metapneumovirus LRTI

**Figure 1.**
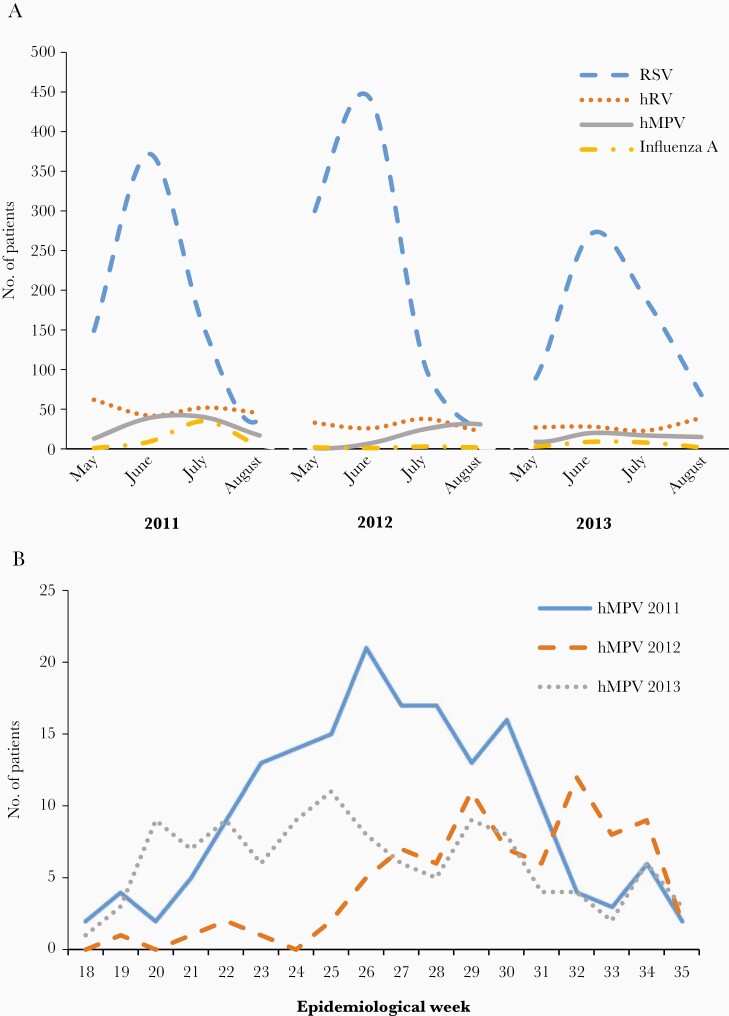
Viral circulation in infants and children 0–24 months of age during the study period. *A*, Number of hospitalized patients with human metapneumovirus (hMPV, solid line) lower respiratory tract illness (LRTI) in comparison to respiratory syncytial virus (RSV, dashed line), human rhinovirus (hRV, dotted line), and influenza A (dash dot line), per study year. *B*, Number of hospitalized patients with hMPV LRTI according to the epidemiological week during the 3 respiratory seasons (2011, solid line; 2012, dashed line; 2013, dotted line).

A total of 4045 hospitalized infants and young children met inclusion criteria; 3947 (98%) agreed to participate in the study. Clinical manifestations on admission were similar for participating and nonparticipating children (not shown). hMPV was detected in 383 (10%) children. Of these, 75 (20% of hMPV infections) were coinfected with RSV, 64 (17%) with hRV, and 1 with influenza A. Six patients were simultaneously infected with hMPV, RSV, and hRV. hMPV cases were more frequent during the first year of the study and always followed the same seasonal pattern as RSV with a distinct midwinter peak (Figure 1). In each year, RSV was the virus most frequently detected in study participants.

**Figure 2.**
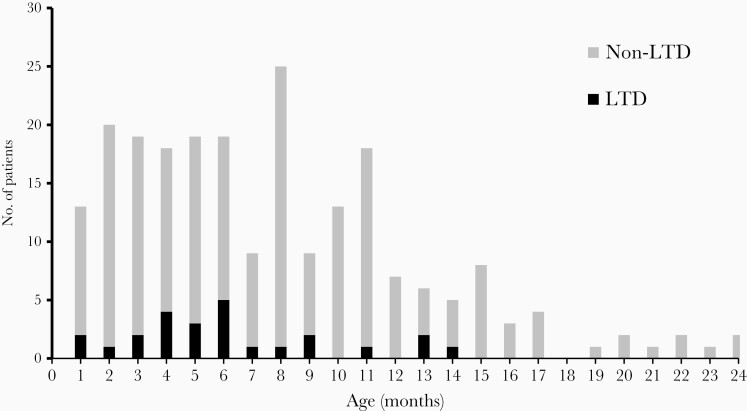
Distribution of life-threatening disease (LTD) in patients with human metapneumovirus (hMPV) severe lower respiratory tract illness according to age. Number of patients with hMPV LTD (LTD; black) or hMPV non LTD (non LTD; gray).

Among the 383 hMPV-infected children, 84% were younger than 1 year with a mean age on admission of 7.4 months (SD, 5.08); 56% were boys. The rate of hospitalization for hMPV LRTI was 3.8 per 1000 infants (95% CI, 3.4–4.2) and 2.26 per 1000 children younger than 2 years (95% CI, 2.04–2.49). Among all hMPV-infected patients, 39 (10.2%) experienced LTD (0.23 per 1000 children; 95% CI, .16–.31/1000). The mean age for LTD was 6.0 months (SD, 3.9; *P* = .08 vs ward admissions) (Figure 2). Two boys died at 3 and 9 months of age due to hMPV, for a case-fatality ratio of 0.52% (95% CI, .06–1.87) and an infant hMPV mortality rate of 0.024 per 1000 (95% CI, .003–.086). Both children had no previously known comorbidities and died due to septic shock and respiratory failure, respectively. Compared to other viral etiologies, even when no virus was isolated, hMPV-associated case-fatality ratio appeared to be lower (RSV, 0.9% [95% CI, .44–1.35)]; hRV, 0.86% [95% CI, .17–2.5]; influenza A, 0.96% ([95% CI, .02–5.2]; and no virus detected, 0.68% [95% CI, .27–1.39]).

Children with hMPV coinfections with RSV, influenza, or hRV experienced more severe clinical signs on admission (tachypnea, tachycardia, wheezing, and chest retractions) than hMPV infections alone. However, coinfections did not increase the risk of LTD (Table 1). In comparison to patients with RSV LRTI, hMPV-infected hospitalized patients were significantly older (OR, 1.05; 95% CI, 1.02–1.07), breastfed less frequently (OR, 0.67; 95% CI, .46–.97), and were at lower risk of experiencing LTD (OR, 0.56; 95% CI, .36–.86) (Table 1).

**Table 1.** Epidemiological and Clinical Differences Between Human Metapneumovirus Single Infection, Human Metapneumovirus Coinfections, and Respiratory Syncytial Virus Single Infection

Variable	hMPV Single Infection (n = 235)	RSV Single Infection (n = 2204)	*P* Value^a^	hMPV Coinfections (n = 148)	*P* Value^b^
Infant variables					
Age, mo, mean (SD)	7.7 (5.04)	6.3 (5.21)	**.0001**	6.9 (5.13)	.119
Male	122 (55.36)	1209 (68.97)	.378	90 (62.5)	.054
Prematurity^c^	34 (14.47)	272 (12.34)	.350	15 (10.14)	.219
Low birth weight^d^	36 (15.32)	269 (12.21)	.171	22 (14.86)	.904
Breastfeeding	199 (84.68)	1967 (89.25)	**.036**	125 (84.46)	.953
Comorbidities^e^	12 (5.11)	86 (3.9)	.373	5 (3.38)	.427
Complete immunization schedule^f^	124 (57.14)	1257 (59.29)	.427	79 (56.83)	.722
Assistance to day care	8 (3.56)	96 (4.47)	.523	6 (4.2)	.755
Malnutrition^g^	2 (1.96)	25 (3.77)	.365	2 (3.13)	.637
Atopy^h^	9 (4.02)	97 (4.55)	.79	3 (2.14)	.296
Familial variables					
Siblings with asthma	33 (19.19)	299 (19.14)	.628	27 (26.47)	.144
Paternal asthma	11 (4.93)	108 (5.3)	.924	6 (4.29)	.364
Maternal asthma	12 (5.38)	118 (5.51)	.279	6 (4.26)	.91
Current maternal smoking	61 (27.6)	561 (26.56)	.818	34 (24.29)	.698
Current paternal smoking	48 (41.38)	637 (45.18)	.672	28 (38.36)	.866
Pregnancy variables					
Intrauterine growth retardation	12 (5.53)	87 (4.15)	.696	11 (7.91)	.761
Teenage mother	22 (9.36)	206 (9.35)	.994	7 (4.73)	.101
Elderly mother	23 (9.79)	257 (11.66)	.392	16 (10.81)	.747
Smoking during pregnancy	46 (20.44)	450 (20.82)	.792	24 (17.02)	.408
Socioeconomic variables					
Crowding	125 (53.19)	1041 (47.23)	.083	78 (52.7)	.926
Precarious home^i^	165 (70.21)	1528 (69.33)	.780	100 (67.57)	.585
Low maternal education^j^	28 (11.91)	295 (13.38)	.528	19 (12.84)	.789
Clinical features at admission					
Tachypnea	57 (24.26)	572 (25.95)	.572	54 (36.49)	**.011**
Tachycardia	53 (22.55)	520 (23.59)	.721	51 (34.46)	**.011**
Wheezing	46 (19.57)	549 (24.91)	.543	43 (29.05)	**.033**
Chest retractions	52 (22.13)	469 (21.28)	.347	50 (33.78)	**.012**
Clinical outcomes during admission					
Pneumonia	21 (8.93)	146 (6.62)	.184	7 (4.73)	.13
Pneumothorax	1 (.43)	17 (.77)	.562	…	…
Sepsis	5 (2.13)	30 (1.36)	.352	1 (.68)	.291
Life-threatening disease^k^	25 (11.11)	370 (18.26)	**.008**	14 (10.14)	.773

Data are No. (%) except where indicated. In bold those P values < .05.

Abbreviations: hMPV, human metapneumovirus; hMPV coinfection, hMPV-positive patient coinfected either with RSV, human rhinovirus, or influenza A viruses; hMPV single infection, hMPV-infected patient without coinfections; RSV, respiratory syncytial virus.

^a^ *P* value: hMPV single infection versus RSV single infection.

^b^ *P* value: hMPV single infection versus hMPV coinfections.

^c^Prematurity: <37 weeks of gestational age.

^d^Low birth weight: <2500 g at birth.

^e^Comorbidities: severe neurologic disorder, congenital cardiopathy, hematologic disorder, or immunodeficiency.

^f^Complete immunization schedule: according to national immunization schedule, https://www.argentina.gob.ar/salud/vacunas.

^g^Malnutrition: % of the infant’s weight compared to that of a normal child (50th percentile of weight for age) of the same age under 90% according to World Health Organization child growth standards: http://www.who.int/childgrowth/standards/en.

^h^Atopy: physician-diagnosed allergic rhinitis or atopic dermatitis.

^i^Precarious home: dirt floor, no sewage, heating unvented sources, lack of potable water, house material tin/mud.

^j^Low maternal education: incomplete primary school.

^k^Life-threatening disease: O_2_ saturation ≤ 87% on admission, requirement for mechanical ventilation, and/or admission to the intensive care unit.

### A Subgroup of Infants at High Risk for Life-threatening hMPV LRTI

**Figure 3.**
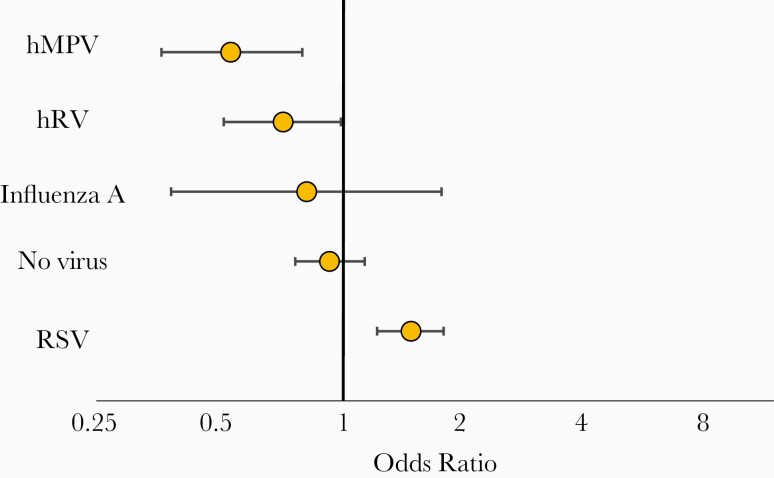
Multivariable analysis of risk for life-threatening disease (LTD) according to infecting virus. Odds ratio with 95% confidence interval for LTD (O_2_ saturation ≤ 87% on admission, requirement for mechanical ventilation, and/or admission to the intensive care unit) in infants and children 0–24 months of age hospitalized for human metapneumovirus single virus or coinfection (hMPV), respiratory syncytial virus (RSV), human rhinovirus (hRV), influenza A single infections, and with no virus detected (no virus) lower respiratory tract illness.

We subsequently explored whether children with hMPV (as a single pathogen or as a component of a coinfection) experienced disease of different severity than children infected with other viruses. Indeed, hMPV LRTI was milder in our population than disease elicited by RSV, hRV, and/or influenza A (OR, 0.53; 95% CI, .36–.79) (Figure 3). But even though disease associated with hMPV was generally milder, a subgroup of infected children still experienced LTD. We therefore investigated whether a subpopulation with identifiable risk factors was disproportionality represented in this subgroup of subjects.

**Figure 4.**
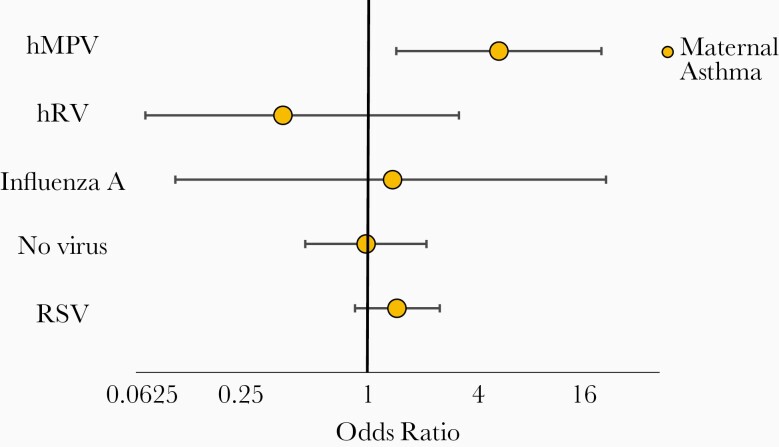
Multivariable analysis of risk for life-threatening disease (LTD) according to infecting virus and maternal asthma status. Odds ratio with 95% confidence interval for LTD (O_2_ saturation ≤ 87% on admission, requirement for mechanical ventilation, and/or admission to the intensive care unit) in infants and children 0–24 months of age hospitalized for human metapneumovirus single virus or coinfection (hMPV), respiratory syncytial virus (RSV), human rhinovirus (hRV), influenza A single infections, and with no virus detected (no virus) lower respiratory tract illness, with maternal asthma.

Given the Th2 polarization in respiratory secretions [16–18] and the reported association between hMPV hospitalizations and asthmatic children [12, 26, 27], we hypothesized that children of asthmatic mothers would be overrepresented among patients infected with the virus experiencing LTD. Indeed, the rate of hMPV LTD in children of asthmatic mothers was 277/1000 compared to children of nonasthmatic mothers whose hMPV LTD rate was 3.4 times lower at 82.3/1000 (OR, 4.29; 95% CI, 1.42–12.93; *P* = .01 for LTD in children of asthmatic mothers vs children born to nonasthmatic mothers). The observed effect for maternal asthma remained significant after adjusting for important confounders (detailed in Table 2; OR, 4.72; 95% CI, 1.39–16.01). Conversely, no increased risk was observed for maternal asthma in promoting LTD during RSV (OR, 1.4; 95% CI, .85–2.33), hRV (OR, 0.36; 95% CI, .07–2.93), or influenza A infections (OR, 1.33; 95% CI, .1–16.91) (Figure 4). Interestingly, when compared with patients with no virus detected as the control group, we observed that children admitted with hMPV infection and whose mothers were not asthmatic had a lowest risk of experiencing LTD (OR, 0.51; 95% CI, .33–.78; interaction *P* value = .024). However, when the mother was asthmatic, hMPV infection did not significantly increase the risk of having LTD compared to those without viral detections (Supplementary Table 1).

**Table 2.** Multivariable Analysis of Risk Factors for Life-threatening Disease in Children Hospitalized With Human Metapneumovirus Infection

Risk Factors	Multivariable Analysis	
	OR (95% CI)	*P* Value
Sewage	0.4 (.14–1.1)	.076
Running water	0.7 (.29–1.72)	.439
Age <6 mo on admission	2.22 (.93–5.27)	.071
Comorbidities^a^	6.47 (1.55–27.00)	.01
Breastfeeding	0.61 (.21–1.77)	.360
Smoking during pregnancy	2.04 (.81–5.14)	.129
Maternal asthma	4.72 (1.39–16.01)	.013
Severe complications^b^	4.69 (1.65–13.37)	.004

Abbreviations: CI, confidence interval; OR, odds ratio.

^a^Comorbidities: severe neurologic disorder, congenital cardiopathy, hematologic disorder, or immunodeficiency.

^b^Severe complications: pneumonia, sepsis, pneumothorax, or apnea.

 Finally, given the rate of hMPV coinfections with RSV in our population (20% of hMPV infections), we explored whether hMPV played an instrumental role in the association between maternal asthma and LTD, or the effect was potentially attributable to effects of RSV. In the subgroup of children with hMPV infections in the absence of RSV (hMPV single infections and coinfections with hRV or influenza A), maternal asthma promoted LTD (OR, 3.8; 95% CI, 1.12–12.88; *P* = .03). The impact of maternal asthma in RSV infections alone was, conversely, not significant.

## Discussion

Our study reveals 2 interesting features of hMPV LRTI. While the virus appears to elicit milder disease among hospitalized children than other agents, among children admitted with hMPV LRTI, those born to asthmatic mothers are at increased risk for LTD. Twenty-eight percent of hMPV-infected patients hospitalized with LRTI and born to asthmatic mothers experienced LTD, in comparison to only 8% hMPV-infected patients hospitalized and born to nonasthmatic mothers. If confirmed in other studies and populations, these observations would be of considerable public health importance, given that asthma is the most frequent chronic disease during pregnancy with rising prevalence, and is now between 10% and 12% [28, 29].

Whether the observed phenomenon is due to direct effects from the immune response or other dysregulations due to maternal asthma during pregnancy [30–32], or represents an early warning in young future asthmatics, needs further investigation. The immune response against hMPV is complex [4]. The virus has been reported to trigger Th2 cytokines through thymic stromal lymphopoietin, IL-4, IL-5, IL-13, and Th-17 responses via the release of IL-1β, IL-17, and IL-6 [17, 18, 33–35]. Interestingly, asthma and pregnancy itself also elicit a Th2 [28, 36, 37] and Th17 [38–40] bias. In studies analyzing other health metrics, maternal asthma has been previously associated with poor maternal (gestational diabetes and placenta previa), perinatal (preterm birth, low birth weight, and preeclampsia), and neonatal (severe hRV LRTI, neonatal death, and hospitalizations) outcomes [28, 29].

As reported by other groups, our study also showed that patients admitted for hMPV LRTI with previous comorbidities and those with severe complications during hospitalization were at higher risk for LTD. These risk factors have been described before for hMPV and other respiratory viruses [3, 12–15].

Several reports describe coinfections between hMPV and other respiratory viruses, mainly with hRV and RSV [41]. As others and our results suggest, hMPV may play an important role in these coinfections [42–44]. Perhaps this phenomenon is explained in part by “viral interference,” when one virus totally or partially blocks the replication of the other pathogen [45]. However, the role for hMPV as a coinfecting agent is still unclear and requires further study [46–48]. In a changing epidemiological landscape driven by preventive interventions against RSV, hMPV may soon alter its role and overall importance as a childhood pathogen should viral replacement occur [49]. Targeting children of asthmatic mothers for prophylaxis against hMPV may be a cost-effective strategy in the era of personalized medicine.

In addition, our study provides important disease burden information about hMPV. The hMPV infant hospitalization rate was surprisingly similar in our population to that in the United States, which is estimated at 2–4.9 per 1000 infants [5, 26], and is considerably lower than that of RSV in other countries [23]. Importantly, the hMPV case fatality ratio in hospital was almost half that reported for RSV in the same population, highlighting that, to date, RSV remains the most important target for prevention [23].

Our study has limitations. First, because (as is the case for most studies) we did not analyze our samples for all other viruses, such as coronavirus or parainfluenza virus type 3, their effect on our observations is unknown. Second, given the age of our subjects and the prospective nature of this program, whether severity depended causally on maternal disease versus infant atopy/asthma cannot be answered. Third, given the observational nature of our study design focused on generating new hypotheses, no adjustment for multiple testing was performed. Additional research is needed to confirm the role of maternal asthma as a risk factor for severity in hMPV-infected children. However, our program also has significant strengths, including its prospective design and the evaluation of respiratory samples using state-of-the-art laboratory RT-PCR techniques. Furthermore, to our knowledge, this is the first population-based study seeking to define the burden of hMPV disease in a vulnerable population from a low- or middle-income country. In addition, this large study addressed numerous prenatal and perinatal risk factors, examining how they modify the severity of infection.

In summary, we report a novel observation that may alter our thinking about severe hMPV infections and, if confirmed in other studies, require us to reformulate its prevention strategies. hMPV represents a threat for life-threatening disease to young children born to asthmatic mothers.

## Supplementary Data

Supplementary materials are available at *The Journal of Infectious Diseases* online. Consisting of data provided by the authors to benefit the reader, the posted materials are not copyedited and are the sole responsibility of the authors, so questions or comments should be addressed to the corresponding author.

jiaa019_suppl_Supplementary_Table

## Notes

INFANT Respiratory Network members: Gustavo Pellegrino, MD, Guadalupe Fernandez Gago, MD, Cecilia Pozzolo, MD, Laura Castro, MD, Rodrigo Egues Almeida, MD, Beatriz Rebec, MD, Mariela Gonzalez, MD, Mariel Calvo, MD, Julieta Henrichsen, MD, Celina Nocito, MD, Guillermo Barbero, MD, Juan Ves Losada, MD, Angel Bonina, MD, Edgardo Flamenco, MD, Alberto Rodriguez Perez, MD, Alicia Kobylarz, MD, Mirta Raggio, MD, Graciela Schavlosky, MD, Adriana Caria, MD, Edgar Barboza, MD, and Gustavo Sastre, MD.

***Financial support.*** This work was supported by the Bill and Melinda Gates Foundation (grant numbers OPP1018614 and OPP1157162 to F. P. P.).

***Potential conflicts of interest.*** All authors: No reported conflicts of interest. All authors have submitted the ICMJE Form for Disclosure of Potential Conflicts of Interest. Conflicts that the editors consider relevant to the content of the manuscript have been disclosed.

Presented in part: 11th International Respiratory Syncytial Virus Symposium, Asheville, NC, 31 October to 4 November 2018; and American Thoracic Society International Conference, Dallas, TX, 17–22 May 2019.
