# Supplementary material for: Role for Maternal Asthma in Severe Human Metapneumovirus Lung Disease Susceptibility in Children
Source: J Infect Dis. 2020 Jan 22;223(12):2072–9. doi: 10.1093/infdis/jiaa019 (PMC7107446; doi:10.1093/infdis/jiaa019)
Supplement: jiaa019_suppl_Supplementary_Table [file jiaa019_suppl_supplementary_table.docx]

| TABLE 1 Epidemiological and clinical differences between human metapneumovirus single infection, human metapneumovirus co-infections and respiratory syncytial virus single infection. | | | | | |
| --- | --- | --- | --- | --- | --- |
|  | **hMPV**  **single infection**  **(n=235)** | **RSV**  **single infection**  **(n=2,204)** | **p-value**^a^ | **hMPV**  **co-infections**  **(n=148)** | **p-value**^b^ |
| ***Infant variables*** |  |  |  |  |  |
| Age, mo, (mean, SD) | 7.7 (5.04) | 6.3 (5.21) | **0.0001** | 6.9 (5.13) | 0.119 |
| Male, n (%) | 122 (55.36) | 1209 (68.97) | 0.378 | 90 (62.5) | 0.054 |
| Prematurity^c^, n (%) | 34 (14.47) | 272 (12.34) | 0.350 | 15 (10.14) | 0.219 |
| Low birth weight^d^, n (%) | 36 (15.32) | 269 (12.21) | 0.171 | 22 (14.86) | 0.904 |
| Breastfeeding, n (%) | 199 (84.68) | 1967 (89.25) | **0.036** | 125 (84.46) | 0.953 |
| Comorbidities^c^, n (%) | 12 (5.11) | 86 (3.9) | 0.373 | 5 (3.38) | 0.427 |
| Complete immunization schedule^f^, n (%) | 124 (57.14) | 1257 (59.29) | 0.427 | 79 (56.83) | 0.722 |
| Assistance to daycare, n (%) | 8 (3.56) | 96 (4.47) | 0.523 | 6 (4.2) | 0.755 |
| Malnutrition^g^, n (%) | 2 (1.96) | 25 (3.77) | 0.365 | 2 (3.13) | 0.637 |
| Atopy^h^, n (%) | 9 (4.02) | 97 (4.55) | 0.79 | 3 (2.14) | 0.296 |
| ***Familial variables*** |  |  |  |  |  |
| Siblings with asthma, n (%) | 33 (19.19) | 299 (19.14) | 0.628 | 27 (26.47) | 0.144 |
| Paternal asthma, n (%) | 11 (4.93) | 108 (5.3) | 0.924 | 6 (4.29) | 0.364 |
| Maternal asthma, n (%) | 12 (5.38) | 118 (5.51) | 0.279 | 6 (4.26) | 0.91 |
| Current maternal smoking, n (%) | 61 (27.6) | 561 (26.56) | 0.818 | 34 (24.29) | 0.698 |
| Current paternal smoking, n (%) | 48 (41.38) | 637 (45.18) | 0.672 | 28 (38.36) | 0.866 |
| ***Pregnancy variables*** |  |  |  |  |  |
| Intrauterine growth retardation, n (%) | 12 (5.53) | 87 (4.15) | 0.696 | 11 (7.91) | 0.761 |
| Teeanage mother, n (%) | 22 (9.36) | 206 (9.35) | 0.994 | 7 (4.73) | 0.101 |
| Elderly mother, n (%) | 23 (9.79) | 257 (11.66) | 0.392 | 16 (10.81) | 0.747 |
| Smoking during pregnancy, n (%) | 46 (20.44) | 450 (20.82) | 0.792 | 24 (17.02) | 0.408 |
| ***Socioeconomic variables*** |  |  |  |  |  |
| Crowding, n (%) | 125 (53.19) | 1041 (47.23) | 0.083 | 78 (52.7) | 0.926 |
| Precarious home^i^, n (%) | 165 (70.21) | 1528 (69.33) | 0.780 | 100 (67.57) | 0.585 |
| Low maternal education^j^, n (%) | 28 (11.91) | 295 (13.38) | 0.528 | 19 (12.84) | 0.789 |
| ***Clinical features at admission*** |  |  |  |  |  |
| Tachypnea, n (%) | 57 (24.26) | 572 (25.95) | 0.572 | 54 (36.49) | **0.011** |
| Tachycardia, n (%) | 53 (22.55) | 520 (23.59) | 0.721 | 51 (34.46) | **0.011** |
| Wheezing, n (%) | 46 (19.57) | 549 (24.91) | 0.543 | 43 (29.05) | **0.033** |
| Chest retractions, n (%) | 52 (22.13) | 469 (21.28) | 0.347 | 50 (33.78) | **0.012** |
| ***Clinical outcomes during admission*** |  |  |  |  |  |
| Pneumonia, n (%) | 21 (8.93) | 146 (6.62) | 0.184 | 7 (4.73) | 0.13 |
| Pneumothorax, n (%) | 1 (0.43) | 17 (0.77) | 0.562 | - | - |
| Sepsis, n (%) | 5 (2.13) | 30 (1.36) | 0.352 | 1 (0.68) | 0.291 |
| Life-threatening disease^k^, n (%) | 25 (11.11) | 370 (18.26) | **0.008** | 14 (10.14) | 0.773 |

hMPV = human Metapneumovirus; hMPV single infection = hMPV infected patients without coinfections; RSV = respiratory syncytial virus; hMPV co-infections = hMPV positive patients co-infected either with RSV, human rhinovirus or influenza A viruses.

^a^p-value: hMPV single infection versus RSV single infection.

^b^p-value: hMPV single infection versus hMPV co-infections.

^c^Prematurity: <37 weeks of gestational age.

^d^Low birth weight: <2500 gr at birth.

^e^Comorbidities: Severe neurologic disorder, congenital cardiopathy, hematologic disorder or immunodeficiency.

^f^Complete immunization schedule: According to National Immunization Schedule, <https://www.argentina.gob.ar/salud/vacunas>.

^g^Malnutrition: % of the infant's weight compared to that of a normal child (50th percentile of weight for age) of the same age under 90% according to World Health Organization child growth standards: http://www.who.int/childgrowth/standards/en

^h^Atopy: Physician diagnosed allergic rhinitis or atopic dermatitis.

^i^Precarious home: Dirt floor; no sewage; heating unvented sources; lack of potable water; house material tin/mud.

^j^Low maternal education: Incomplete primary school.

^k^Life-threatening disease: O2 sat≤ 87% on admission, requirement for mechanical ventilation and/or admission to the intensive care unit.

| \| **TABLE 2.**  Multivariable analysis of risk factors for life-threatening disease in children hospitalized with hMPV infection. \| \| \| \| --- \| --- \| --- \| \|  \| **Multivariable analysis** \| \| \|  \| OR (CI 95%) \| p-value \| \| Sewage \| 0.4 (0.14-1.1) \| 0.076 \| \| Running water \| 0.7 (029-1.72) \| 0.439 \| \| <6 months on admission \| 2.22 (0.93-5.27) \| 0.071 \| \| **Comorbidities^a^** \| 6.47 (1.55-27) \| 0.01 \| \| Breastfeeding \| 0.61 (0.21-1.77) \| 0.360 \| \| Smoking during pregnancy \| 2.04 (0.81-5.14) \| 0.129 \| \| **Maternal asthma** \| 4.72 (1.39-16.01) \| 0.013 \| \| **Severe complications^b^** \| 4.69 (1.65-13.37) \| 0.004 \|   OR indicates odds ratio; CI, confidence interval.  ^a^Comorbidities: Severe neurologic disorder, congenital cardiopathy, hematologic disorder or immunodeficiency.  ^b^Severe complications: Pneumonia, sepsis, pneumothorax or apnea. |
| --- | --- | --- | --- | --- | --- | --- | --- | --- | --- | --- | --- | --- | --- | --- | --- | --- | --- | --- | --- | --- | --- | --- | --- | --- | --- | --- | --- | --- | --- | --- | --- | --- | --- |
